# Long-term safety and immunologic outcomes of daily oral immunotherapy for peanut allergy

**DOI:** 10.1016/j.jacig.2023.100120

**Published:** 2023-05-27

**Authors:** J. Andrew Bird, Caroline Nilsson, Kari Brown, Trinh Pham, Stephen Tilles, George du Toit, Amal Assa’ad

**Affiliations:** a Department of Pediatrics, University of Texas Southwestern, Dallas, Tex; b Clinical Research and Education, Karolinska Institutet, Sachs’ Children and Youth Hospital, Stockholm, Sweden; c Aimmune Therapeutics, Brisbane, Calif; d Guy’s and St Thomas’ NHS Foundation Trust, London, United Kingdom; e Division of Allergy and Immunology, Cincinnati Children’s Hospital Medical Center, Cincinnati, Ohio

**Keywords:** PTAH, oral immunotherapy, peanut allergy, peanut (*Arachis hypogaea*) allergen powder, pooled safety, immunologic outcomes

## Abstract

**Background:**

Oral immunotherapy containing peanut (*Arachis hypogaea*) allergen powder-dnfp (PTAH) (Palforzia [Aimmune Therapeutics, Brisbane, Calif]) for 9 to 12 months resulted in higher tolerated amounts of peanut protein in PTAH-treated individuals aged 4 to 17 years with peanut allergy than in placebo-treated participants.

**Objective:**

We aimed to describe additional long-term pooled safety data and changes in peanut sensitization markers from baseline through approximately 5 years of treatment.

**Methods:**

The results from 6 clinical trials of PTAH (3 controlled and 3 open-label extension studies [N = 1227]) were pooled, and analysis of safety outcomes and immunologic data was performed. The PTAH doses were administered sequentially as follows: initial dose escalation (dose increased to 6 mg over 2 days), updosing (dose increased every 2 weeks to 300 mg for a minimum of 6 months), and maintenance dosing (300 mg per day).

**Results:**

There was a trend toward decreased adverse events (AEs) at years 1 and 2 that was maintained up to 5 years, with 94% of patients experiencing mild or moderate AEs and only 13% discontinuing PTAH use because of AEs overall. Gastrointestinal symptoms were the most commonly reported treatment-related AEs. A downward trend in systemic allergic reactions was also reported. PTAH treatment resulted in reduced levels of peanut-specific IgE after the first year and increased levels of peanut-specific IgG4, with a lowered peanut-specific IgE:IgG4 ratio. A reduction in median peanut skin prick test wheal diameter was observed (11.50 mm at baseline vs 5.75 mm at year 5).

**Conclusion:**

Long-term immunomodulation without any new safety signals was reported with PTAH immunotherapy in the largest safety data set and longest treatment duration for oral immunotherapy published to date.

## Introduction

Peanut (*Arachis hypogaea*) allergen powder-dnfp (PTAH [Palforzia, Aimmune Therapeutics, Brisbane, Calif]), a peanut oral immunotherapy, is approved for mitigation (when used in combination with a peanut avoidance diet) against allergic reactions after accidental exposure to peanuts in individuals aged 4 to 17 years with a confirmed peanut allergy.^1^^,^^2^

In clinical trials, PTAH-treated children and teenagers with peanut allergy tolerated higher amounts of peanut protein than did placebo-treated participants after 9 to 12 months of treatment.^3^^,^^4^ A pooled safety analysis of 3 phase 3 randomized, double-blind, placebo-controlled, multicenter trials (PALISADE,^3^ RAMSES, and ARTEMIS^4^) and 2 uncontrolled, open-label extension safety trials^5^ has recently been published; in the pooled analysis, PTAH demonstrated a consistent and manageable safety profile that improved over time.^6^

In this Brief Report, we describe additional, long-term, pooled safety data for up to approximately 5 years and changes in peanut sensitization markers from baseline through approximately 5 years of treatment with PTAH. The results from 6 PTAH clinical trials (3 controlled trials [NCT02635776, NCT03126227, and NCT03201003; see Table E1 in the Online Repository at www.jaci-global.org] and 3 open-label extension studies [NCT02993107, NCT03292484, and NCT03337542]) were pooled, and analysis of safety outcomes and immunologic data was performed (see Fig E1 in the Online Repository at www.jaci-global.org). The ARC008 open-label extension study (NCT03292484) is currently ongoing; thus, this update (data cutoff date July 31, 2021) represents an interim data cut in advance of the completion of that study.

PTAH treatment was administered across 3 sequential daily dosing phases: initial dose escalation (in clinic), with the dose increased to 6 mg over 2 days; updosing, with the dose increased every 2 weeks to 300 mg for a minimum of 6 months; and maintenance dosing of 300 mg per day.

Adverse events (AEs) were recorded by study staff during in-clinic dosing and by participants and/or caregivers for those events occurring outside the clinic, with the relationship of AEs to study treatment determined by investigators. The Common Toxicity Criteria for Adverse Events (version 4.03)^7^ were used by investigators to assess the severity of all AEs barring hypersensitivity events, in which case Consortium of Food Allergy Research–modified Common Toxicity Criteria for Adverse Events were applied (see Table E2 in the Online Repository at www.jaci-global.org).^8^ Systemic allergic reactions (SARs)—anaphylactic reactions of any severity, as defined by the Sampson criteria^9^—were graded using the 3-point Muraro grading scale (see Table E3 in the Online Repository at www.jaci-global.org).^10^

Blood samples were obtained at prespecified time points to analyze changes in peanut-specific IgE (psIgE) level, peanut-specific IgG4 (psIgG4) level, and the ratio of these (psIgE:psIgG4), as well as changes in peanut skin prick test (SPT) wheal diameter.

For detailed methods, please see the Methods section in this article’s Online Repository at www.jacionline.org.

## Results and discussion

A total of 1127 patients were included in the pooled analysis. Demographics and baseline characteristics of the pooled study population (data cutoff date July 31, 2021) are shown in Table I. The median treatment duration at the time of data analysis was 2.3 years (interquartile range 0.86-3.6 years) with a maximum duration of 5.5 years.

**Table I:** Participant demographics and baseline characteristics

Baseline characteristic	Pooled population (N = 1127)∗
Age (y), median (range)	9.0 (4-17)
Age category (y), no. (%)	
4-11	753 (66.8)
12-17	374 (33.2)
Sex, no. (%)	
Male	675 (59.9)
Female	452 (40.1)
Race, no. (%)	
White	848 (75.2)
Other	268 (23.8)
History of allergic rhinitis, no. (%)†	785 (69.7)
History of asthma, no. (%)†	551 (48.9)
History of atopic dermatitis, no. (%)†	666 (59.1)
Other food allergies, no. (%)†	709 (62.9)
Peanut SPT average wheal diameter (mm)	
No. of patients	1075
Median (Q1, Q3)	11.5 (9.0, 15.5)
Peanut-specific IgE level (kUA/L)	
No. of patients	937
Median (Q1, Q3)	83.9 (30.0, 205.0)

*Q*, Quartile.

∗ Baseline data were taken from the trial in which a participant was first treated with PTAH.

† Participants may be included in more than 1 category.

**Fig 1:**
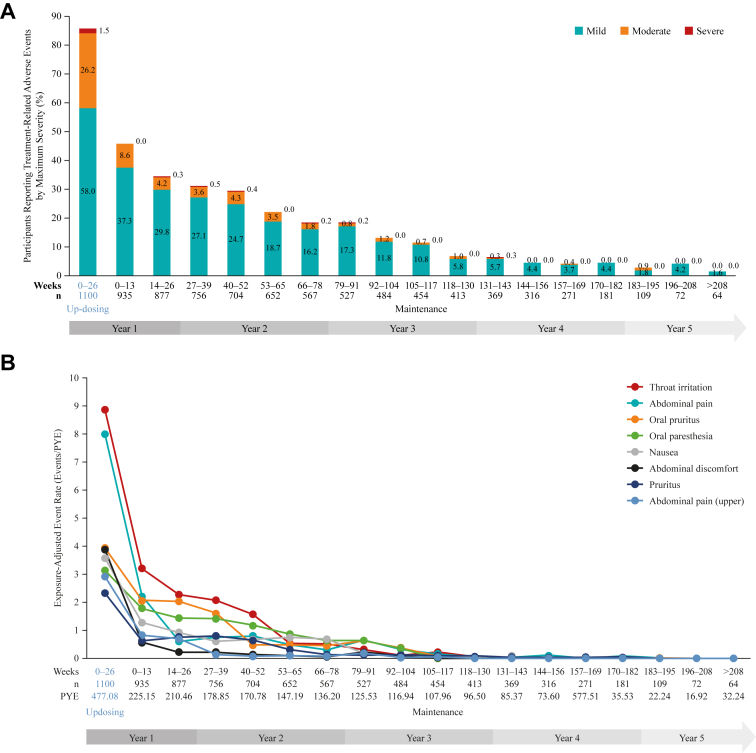
**A** and **B,** Percentage of participants reporting any treatment-related AEs by maximum severity during treatment with PTAH (**A**) and commonly reported treatment-related adverse events adjusted for exposure (**B**).

**Fig 2:**
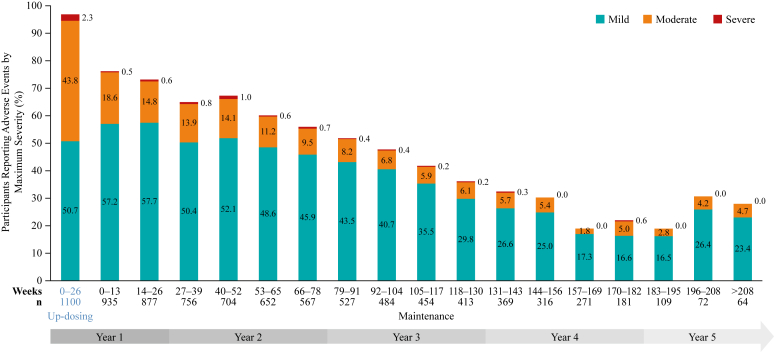
Percentage of participants reporting adverse events by maximum severity during treatment with PTAH.

**Fig 3:**
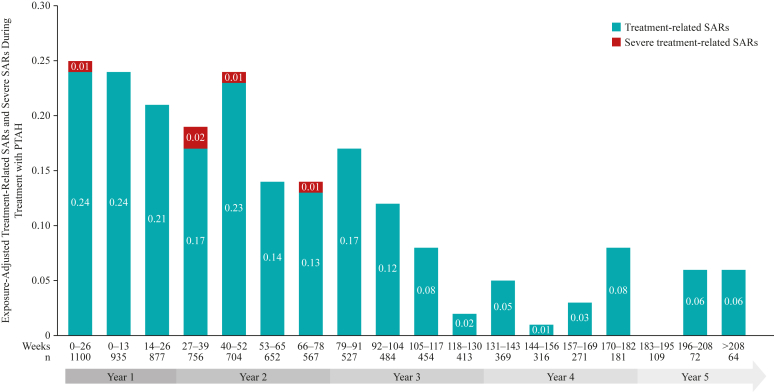
Exposure-adjusted event rates of treatment-related SARs and severe SARs during treatment with PTAH.

In the safety population (subjects aged 4-17 years who were receiving at ≥1 dose of PTAH), most participants (n = 935 [83.0%]) were able to reach the 300-mg PTAH dose level. Consent withdrawal (n = 363 [32.2%]) was the most common primary reason for treatment discontinuation. Safety data were examined out to approximately 5 years’ follow-up, with 90.6% of patients (n = 1021) experiencing at least 1 treatment-related AE (TRAE). TRAEs were most common in the updosing period, representing the first 6 months of treatment (Fig 1, *A*), and they were predominantly graded as mild (n = 638 [58.0%]) or moderate (n = 288 [26.2%]) in severity, with 17 participants (1.5%) experiencing a maximum TRAE grade of severe. In total, serious AEs were reported by 44 participants (3.9%), whereas serious AEs considered related to treatment occurred in 7 participants (0.6%). Severe or life-threatening AEs occurred in 62 participants (5.5%); overall, 149 participants (13.2%) discontinued PTAH on account of AEs. A total of 16 cases of eosinophilic esophagitis were diagnosed during the study period; details on suspected and diagnoses cases of eosinophilic esophagitis captured during follow-up through to December 15, 2018, have been reported in detail previously.^11^ When all AEs during updosing were considered, maximum severity was predominantly mild (n = 558 [50.7%]) or moderate (n = 482 [43.8%]), and rates of AEs remained low during the extended follow-up (Fig 2). Exposure-adjusted rates of treatment-related SARs of any severity trended downward throughout follow-up, and no severe treatment-related SARs occurred beyond year 2 (Fig 3). The most frequently reported TRAEs, adjusted for exposure, were primarily respiratory (eg, throat irritation) or gastrointestinal (eg, abdominal pain, oral pruritus, nausea), and they occurred at the highest level during the updosing phase before declining through the long-term follow-up phase (Fig 1, *B*).

**Fig 4:**
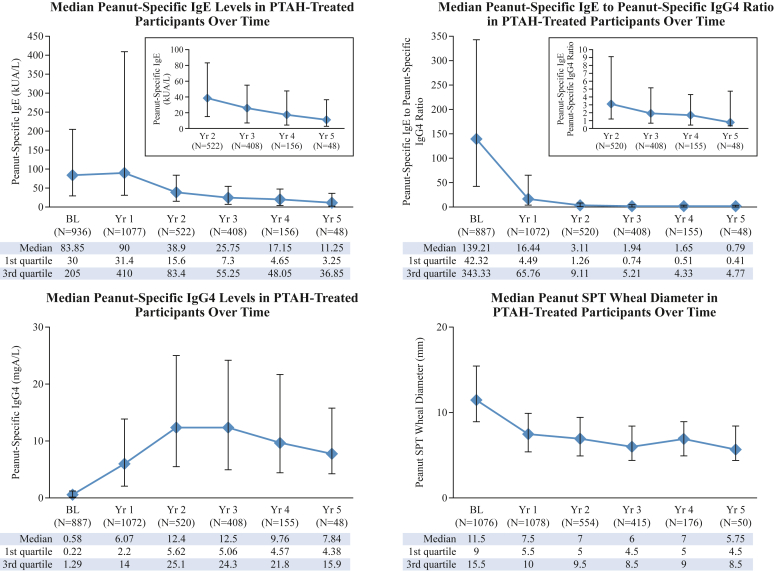
Immunologic end points following long-term treatment with PTAH. Values are median peanut-specific immunoglobulin levels and SPT wheal diameters, with error bars representing first and third quartile values. *BL*, Baseline; *Ig*, immunoglobulin.

Those safety population subjects with at least 1 immunologic data point were included in the immunologic analysis. The median psIgE level was 83.9 kUA/L at baseline and declined in year 2 (38.9 kUA/L), further decreasing with continued treatment over time, whereas the median psIgG4 level increased to a peak around years 2 to 3 (approximately 12.5 mgA/L [Fig 4]). The psIgE:psIgG4 ratio between these 2 parameters declined substantially in year 1 (baseline median 139.21; year 1 median 16.44) and remained low during treatment exposure. The median peanut SPT wheal diameter was 11.50 mm at baseline and decreased over time to 5.75 mm at year 5 (Fig 4).

This updated, pooled analysis of PTAH in individuals aged 4 to 17 years with peanut allergy represents the largest safety data set and the longest treatment duration published to date, with approximately 2300 patient exposure years of PTAH evaluated. The results from these long-term pooled analyses should be interpreted cautiously on account of differences in study designs, geographic locations, and treatment durations between trials, with the potential for survival bias observed with *post hoc* analyses involving open-label studies. Further analyses of these data sets will also include the final data from studies that are ongoing, and thus these results may not necessarily reflect the completed study outcomes. In addition, sample sizes were smaller over time; however, as the NCT03292484 trial continues, more participants will receive PTAH for a longer duration, allowing for a larger sample with at least 5 years of treatment.

The safety data demonstrated a trend toward decreased AEs at years 1 and 2 that appears to be maintained with a follow-up of up to 5 years, with no new safety signals uncovered. Most patients (94%) experienced a maximal AE severity graded as mild or moderate, and only 13% discontinued treatment on account of AEs. Gastrointestinal symptoms (eg, abdominal pain, vomiting, nausea) were among the most commonly reported TRAEs, which is consistent with reports from other oral immunotherapy studies.^12^, ^13^, ^14^ Gastrointestinal symptoms were also the most common AEs leading to discontinuation.^6^ In addition, SARs generally trended downward as treatment continued. SARs during oral immunotherapy are a known risk of PTAH.^15^ In this analysis, and consistent with previous analyses, these reactions commonly occurred within 2 hours of dosing, and most individuals who experienced a SAR had only a single event and chose to continue treatment. These data support a trend in decreased frequency of allergic reactions with long-term PTAH treatment. Additionally, the risk of a severe systemic reaction was low, and the majority of these individuals also chose to continue treatment after the event. The safety data presented here are aligned with the findings of Brown et al,^6^ and they provide further supporting evidence for clinicians, patients, and their families when considering the tolerability of long-term treatment with oral immunotherapy for peanut allergy.

Treatment with PTAH for up to approximately 5 years induced long-term immunomodulation, with analysis of immunologic parameters revealing that over time, PTAH treatment resulted in reduced psIgE levels after the first year and increased psIgG4 levels, with a lowered psIgE:psIgG4 ratio and reduced peanut SPT wheal diameter. These changes in immunologic end points align with the trend toward decreasing AE frequency and severity, and they are consistent with the known immunologic mechanisms of oral immunotherapy,^16^ resulting in favorable changes in peanut-specific immunologic markers that have been previously reported with PTAH.^3^

The clinical course of peanut allergy can be lifelong, with accidental exposure to peanut relatively common, representing an ongoing challenge and affecting day-to-day life for patients and their families.^17^ Thus, a comprehensive safety evaluation of more patient years of drug exposure with long-term use of therapies for peanut allergy is imperative and will enable clinicians and patients to be better informed of the safety profile with prolonged use. The demonstration of long-term immunologic effects of oral immunotherapy in these patients provides additional context to the safety findings and previously reported data for PTAH.

The results of this long-term, pooled analysis can be used to facilitate shared discussions involving all stakeholders to help ensure that appropriate clinical treatment decisions are made. Although these results should be interpreted with caution and (at this stage) no predictors of AEs can be identified, they suggest that patients who continue treatment beyond 2 years experience fewer AEs over time and that their long-term immunomodulatory biomarker profile resembles those seen with other forms of successful allergen immunotherapy.

## Disclosure statement

Supported by Aimmune Therapeutics, a Nestlé Health Science company, which designed and sponsored the clinical trials and analyses described in addition to providing financial support for the development of this article. There were no agreements between the sponsor and the authors or their institutions, which treated the trial data as confidential information of the sponsor.

Disclosure of potential conflict of interest: J. A. Bird reports research grants from Aimmune Therapeutics, Astellas, DBV Technologies, Food Allergy Research and Education (FARE), Genentech, the National Institute of Allergy and Infectious Diseases of the National Institutes of Health (NIH), Novartis, and Regeneron, as well as consulting fees from AllerGenis, Allergy Therapeutics, Before Brands, DBV Technologies, FARE, HAL Allergy, Novartis, and Prota. C. Nilsson reports grants to her institution and advisory board fees from Aimmune Therapeutics and Novartis, as well as lecture fees from MEDA, ALK, Thermo Fisher, and GSK. K. R. Brown is an employee of Aimmune Therapeutics and was a subinvestigator in Aimmune Therapeutics clinical studies before her employment. She has a patent pending (US 16/721805). T. Pham and S. Tilles are employees of Aimmune Therapeutics. G. du Toit reports research grants to his institution and advisory board fees from Aimmune Therapeutics. A. Assa’ad reports research grants from NIH, FARE, AbbVie, DBV Technologies, Novartis, AstraZeneca, Alladapt, Sanofi Aventis, Siolta, and Regeneron, and speaker fees from Peerview; in addition, she has a patent (US 7,732,135 B2).

Ethics committee approval: Approvals were obtained from independent ethics committees. All of the participants, or a parent or guardian, provided written informed consent. Minor children, who were not of legal age to provide consent, provided assent in accordance with the applicable age or based on local regulatory requirements.

Data sharing: All relevant data are included within the article and its supplementary files (available in the Online Repository at www.jaci-global.org). Deidentified participant data (including data dictionaries) will not be shared. PTAH (Palforzia), the first oral biologic immunotherapy, is approved by the US Food and Drug Administration and European Commission and is currently being considered by other regulatory authorities. Additional data requests or clarifications can be made to the corresponding author.

**Clinical implications**

The results of this study can be used to facilitate shared clinical discussions involving all stakeholders to help ensure that appropriate PTAH treatment decisions are made for patients with peanut allergy.

## Supplementary data

Supplementary Fig E1
