# Supplementary figures and images for: Long-term safety and immunologic outcomes of daily oral immunotherapy for peanut allergy
Source: J Allergy Clin Immunol Glob. 2023 May 27;2(3):100120. doi: 10.1016/j.jacig.2023.100120 (PMC10509957; doi:10.1016/j.jacig.2023.100120)

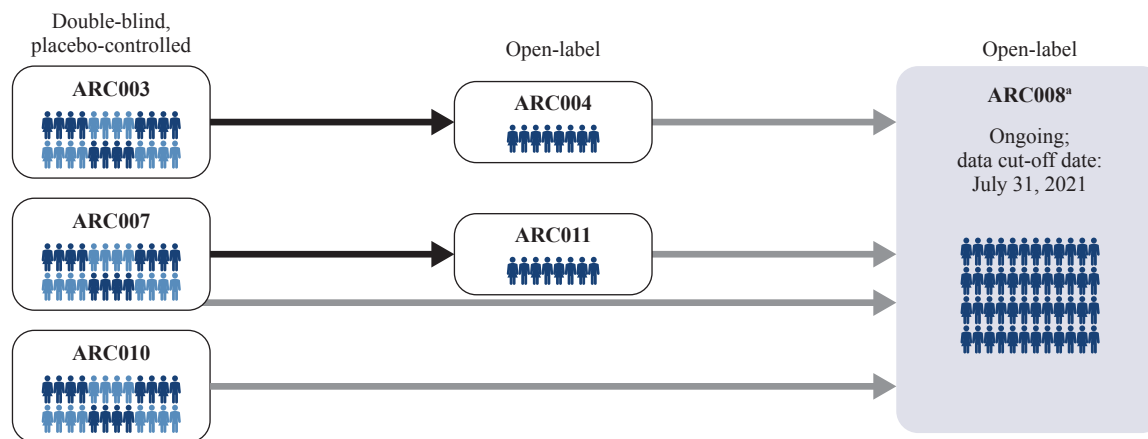

Supplement: Supplementary Fig E1 [file mmc1.pdf]
