# Supplementary material for: Long-term safety and immunologic outcomes of daily oral immunotherapy for peanut allergy
Source: J Allergy Clin Immunol Glob. 2023 May 27;2(3):100120. doi: 10.1016/j.jacig.2023.100120 (PMC10509957; doi:10.1016/j.jacig.2023.100120)
Supplement: Supplementary Materials [file mmc2.docx]

# Online Repository

# Methods

## Dosing Regimen

Participants initiated treatment with a dose of 0.5 mg. The dose was increased incrementally, as tolerated, at 20- to 30-minute intervals over the course of a single day to a maximum dose of 6 mg. If at least 3 mg was tolerated on Day 1, a single 3-mg dose was given on Day 2. Product doses were 0.5, 1, 1.5, 3, and 6 mg.

Participants were given escalating doses from 3 to 300 mg/day at 2-week intervals, as tolerated. This up-dosing phase lasted approximately 20 weeks up to a maximum of 40 weeks. Product doses were 3, 6, 12, 20, 40, 80, 120, 160, 200, 240, and 300 mg (maintenance dose).

Dose adjustments and interruptions were permitted based upon pre-specified criteria that incorporated the clinical judgment of the investigator. To fully evaluate the adverse event (AE) profile, prophylactic antihistamines were generally not allowed; however, medications used to manage atopic conditions were allowed and collected in the database as concomitant medications. Although alternative non-daily dosing regimens were evaluated in ARC004, this analysis focuses on daily dosing only. In conjunction with the trial treatment’s approved indication, all trials required participants to continue a peanut-avoidant diet and to carry an epinephrine auto-injector.

## Clinical Trials

Safety data were obtained from three completed phase 3 trials and two completed open-label extension trials of peanut (*Arachis hypogaea*) allergen powder-dnfp (PTAH) conducted in Europe and North America between January 8, 2016 and July 31, 2021 (PALISADE [NCT02635776]; RAMSES [NCT03126227]; ARTEMIS [NCT03201003]; PALISADE follow-on study [NCT02993107]; RAMSES follow-on study [NCT03337542]), as well as one ongoing open-label study (ARC008 [NCT03292484]). Safety of PTAH in a pre-specified safety population of children and teenagers (aged 4–17 years) with a confirmed diagnosis of peanut allergy was a key endpoint in all trials.

All trial designs were approved by institutional review boards at participating sites and were conducted in accordance with the International Conference on Harmonization Guidelines for Good Clinical Practice and the Declaration of Helsinki and its amendments. All participants provided written informed consent or assent (as appropriate for children) before participation.

## Participant Eligibility

Key eligibility criteria for each controlled trial are detailed in Figure E1 and Table E1. PALISADE enrolled participants (aged 4–55 years) from North America and Europe based on clinical history of peanut allergy, confirmatory skin prick test (SPT; SPT wheal ≥3 mm compared with negative saline control) and/or elevated peanut-specific immunoglobulin E (psIgE; ≥0.35 kUA/L), and dose-limiting symptoms to ≤100-mg peanut protein at screening double-blind, placebo-controlled food challenge (DBPCFC),^1^ following PRACTALL guidelines.^2^ All placebo-treated participants who completed PALISADE, and PTAH-treated participants who tolerated at least the 300-mg peanut protein dose at exit DBPCFC, were eligible to enter the follow-on trial (ARC004). Key eligibility criteria for the ARTEMIS trial were identical to PALISADE, with the exception of a higher maximum reaction threshold at screening DBPCFC (300-mg peanut protein), and the age range (4–17 years) and geography (Europe only) of individuals screened.^3^ RAMSES enrolled participants aged 4–17 years from North America who met more stringent peanut allergy clinical history and peanut sensitization criteria (SPT wheal ≥8 mm compared with negative saline control; psIgE ≥14 kUA/L), and did not undergo DBPCFC at screening. RAMSES participants who received active treatment and completed the trial were eligible to enter the follow-on trial (ARC011). All placebo-treated participants who completed RAMSES or ARTEMIS, and PTAH-treated participants who completed studies ARC004, ARC011, or ARTEMIS, were eligible to enter the longer-term follow-on trial ARC008.

## Trial Populations

Safety data and immunological analyses include all participants, aged 4–17 years, who received at least one dose of active drug following a once-daily dosing regimen across any of the six trials and excluded placebo data, with immunological endpoints evaluated in patients who had at least one immunological data point at the time of data cut-off included in the immunological analysis.

## Assessments and Analyses

The visit schedule was similar between the trials: during up-dosing (approximately 6 months), patients had scheduled visits every two weeks; during the following 6 months (the first 6 months of maintenance treatment), patients had scheduled visits every month; during the rest of the follow-up period, visits were scheduled every 3 months. Unscheduled visits were available to the families at any time. Communication between the families and investigators was extensive and fluid during the trials. The families had the phone numbers and emails of the sites, which they could use as needed. They were instructed to communicate with the sites immediately in case they had AEs of special interest or if they had any concern or question. AEs of special interest included systemic allergic reactions (as defined below), epinephrine use, accidental and nonaccidental food allergen exposure (with or without symptoms), serious adverse events, chronic GI AEs, and pregnancy. The request for unscheduled visits could be done by phone, email, as preferred.

Reported AEs were summarized using the MedDRA System Organ Class and Preferred Term, and data were presented as numbers and percentages of participants or events. Attribution of the treatment-relatedness of AEs was performed by the investigators. For patients with chronic or recurrent gastrointestinal symptoms, investigators were advised to have a low threshold of instituting dose modifications, could refer participants to an outside specialist at their discretion or obtain additional testing, and were provided guidance on when to refer to a gastroenterologist due to the potential for eosinophilic esophagitis.

Given differences in trial designs and extent of treatment exposure, no formal inferential statistical comparisons have been conducted; safety data are summarized using appropriate descriptive statistics. Exposure-adjusted incidence rates are defined as the total number of events divided by the total number of participant-years at risk (PYE); the total number of PYE is defined as the total number of days on treatment for all participants divided by 365.25.

## Defining Adverse Events

Common Toxicity Criteria for Adverse Events (CTCAE; v4.03) were used by investigators to assess the severity of all AEs barring hypersensitivity events where Consortium of Food Allergy Research-modified CTCAE were applied (Table E2). Systemic allergic reactions were graded using the 3-point Muraro grading scale (Table E3).^4^ Across this analysis, a “systemic allergic reaction” refers to an anaphylactic reaction event of any severity, as defined according to National Institute of Allergy and Infectious Disease and Food Allergy and Anaphylaxis Network criteria;^5^ “anaphylaxis” was used to distinguish a systemic allergic reaction event that was graded as severe on the Muraro grading scale. Epinephrine use was analyzed by episode, defined as the administration of one or more epinephrine doses within 2 hours of the occurrence of the AE.

*AE:* any untoward medical occurrence that occurred during the conduct of the study, regardless of relationship to study product. Any change in clinical status, routine laboratory test results, and physical examinations that was considered clinically significant by the investigator was considered an AE.

*Serious adverse event:* an AE that resulted in any of the following: death; life-threatening (the subject was at immediate risk of death from the reaction as it occurred, in the opinion of the investigator or sponsor); inpatient hospitalization or prolongation of existing hospitalization (hospitalization only for observation was not considered a serious AE); persistent or significant incapacity or substantial disruption of the ability to conduct normal life functions; congenital abnormality or birth defect; important medical event that may have not resulted in one of the above outcomes, but may have jeopardized the health of the subject or required medical or surgical intervention to prevent one of the outcomes listed in the definition of serious event.

*Systemic allergic reaction:* all events that mapped to the MedDRA preferred term of “anaphylactic reaction” regardless of severity (mild, moderate, severe) or trigger (study product, food allergen, other allergen). Term “anaphylaxis” was used to describe the subset of systemic allergic reactions that were severe (eg, severe systemic allergic reactions [anaphylaxis]).

*Anaphylaxis:* anaphylaxis (MedDRA preferred term “anaphylactic reaction”) was defined as a severe, potentially life-threatening, systemic hypersensitivity reaction characterized by rapid onset with life-threatening airway, breathing, or circulatory problems that usually, although not always, are associated with skin and mucosal changes. In these analyses, the term “anaphylaxis” was used only to describe a subset of systemic allergic reactions that were considered severe (grade 3) using the 3‑point Muraro grading scale.

# References

1. Vickery BP, Vereda A, Casale TB, Beyer K, du Toit G, Hourihane JO, et al. AR101 Oral Immunotherapy for Peanut Allergy. N Engl J Med 2018;379:1991-2001.

2. Sampson HA, Gerth van Wijk R, Bindslev-Jensen C, Sicherer S, Teuber SS, Burks AW, et al. Standardizing double-blind, placebo-controlled oral food challenges: American Academy of Allergy, Asthma & Immunology-European Academy of Allergy and Clinical Immunology PRACTALL consensus report. J Allergy Clin Immunol 2012;130:1260-1274.

3. Hourihane JOB, Beyer K, Abbas A, Fernández-Rivas M, Turner PJ, Blumchen K, et al. Efficacy and safety of oral immunotherapy with AR101 in European children with a peanut allergy (ARTEMIS): a multicentre, double-blind, randomised, placebo-controlled phase 3 trial. Lancet Child Adolesc Health 2020;4:728-739.

4. Muraro A, Roberts G, Clark A, Eigenmann PA, Halken S, Lack G, et al. The management of anaphylaxis in childhood: position paper of the European academy of allergology and clinical immunology. Allergy 2007;62:857-871.

5. Sampson HA, Munoz-Furlong A, Campbell RL, Adkinson NF, Jr., Bock SA, Branum A, et al. Second symposium on the definition and management of anaphylaxis: summary report--Second National Institute of Allergy and Infectious Disease/Food Allergy and Anaphylaxis Network symposium. J Allergy Clin Immunol 2006;117:391-397.

Tables

## ***Table*** E1. Summary of randomized controlled trials

|  | **PALISADE** | **RAMSES** | **ARTEMIS** |
| --- | --- | --- | --- |
| **Planned and actual enrollment, n** | Planned: N~500; actual: N=555 | Planned: N=500; actual: N=506 | Planned: N=160; actual: N=175 |
| **Treated and completed, n** | Treated: N=551  (n=413, PTAH; n=138, PBO)  Completed: N=442  (n=314, PTAH; n=128, PBO) | Treated: N=505  (n=337, PTAH; n=168, PBO)  Completed: N=418  (n=260, PTAH; n=158, PBO) | Treated: N=175  (n=132, PTAH; n=43, PBO)  Completed: N=146  (n=106, PTAH; n=40, PBO) |
| **Key inclusion criteria** | | | |
| Age | 4–55 years (primary analysis: 4–17 years) | 4–17 years | 4–17 years |
| Peanut allergy history | Clinical history of allergy to peanut or peanut-containing foods | History of physician-diagnosed IgE-mediated peanut allergy, which includes the onset of characteristic allergic signs and symptoms within 2 hours of known oral exposure to peanut or peanut-containing foods^a^ | Clinical history of allergy to peanut or peanut-containing foods |
| Serum psIgE | ≥0.35 kUA/L^b^ | ≥14 kUA/L | ≥0.35 kUA/L^b^ |
| Mean peanut wheal diameter on SPT | ≥3 mm compared with negative saline control^b^ | ≥8 mm compared with negative saline control | ≥3 mm compared with negative saline control^b^ |
| Screening DBPCFC | DLS ≤100-mg peanut protein | Not performed | DLS ≤300-mg peanut protein |
| **Key exclusion criteria** | | | |
| Prior history | Cardiovascular disease, including uncontrolled or inadequately controlled hypertension; severe or life-threatening episode of anaphylaxis or anaphylactic shock within 60 days of screening; EoE; other eosinophilic gastrointestinal disease; chronic, recurrent, or severe GERD; symptoms of dysphagia or recurrent gastrointestinal symptoms of undiagnosed etiology; mast cell disorder, including mastocytosis; urticarial pigmentosa, chronic idiopathic, or chronic physical urticaria beyond simple dermatographism (eg, cold urticaria, cholinergic urticaria); and hereditary or idiopathic angioedema | | |

^a^Participants for whom the clinical diagnosis of peanut allergy is uncertain were excluded.
^b^Participants must meet serum psIgE **and/or** SPT criteria for inclusion in PALISADE and ARTEMIS.
DBPCFC, double-blind, placebo-controlled food challenge; DLS, dose-limiting symptoms; EoE, esophagitis; GERD, gastroesophageal reflux disease; IgE, immunoglobulin E;
PBO, placebo; psIgE, peanut-specific immunoglobulin E; PTAH, peanut (Arachis hypogaea) allergen powder-dnfp; SPT, skin prick test.

## ***Table*** E2. Consortium of Food Allergy Research-modified Common Toxicity Criteria for Adverse Events (CTCAE)

| **Grade 1 Mild** | **Grade 2 Moderate** | **Grade 3 Severe** | **Grade 4 Life-threatening** | **Grade 5**  **Death** |
| --- | --- | --- | --- | --- |
| Transient or mild discomforts (<48 hours), no or minimal medical intervention/therapy required. These symptoms may include pruritus, swelling or rash, abdominal discomfort or other transient symptoms. | Symptoms that produce mild to moderate limitation in activity, some assistance may be needed; no or minimal intervention/ therapy is required. Hospitalization is possible. These symptoms may include persistent hives, wheezing without dyspnea, abdominal discomfort/increased vomiting, or other symptoms | Marked limitation in activity, some assistance usually required; medical intervention/therapy required, hospitalization is possible. Symptoms may include bronchospasm with dyspnea, severe abdominal pain, throat tightness with hoarseness, transient hypotension, among others. Parenteral medication(s) usually indicated. | Extreme limitation in activity, significant assistance required; significant medical/therapy. Intervention is required; hospitalization is probable. Symptoms may include persistent hypotension and/or hypoxia with resultant decreased level of consciousness associated with collapse and/or incontinence or other life-threatening symptoms. | Death. |

## ***Table*** E3. Muraro/EAACI scale for grading systemic allergic reactions

| **Grade** | **Defined by** |
| --- | --- |
| 1. *Mild* (skin and subcutaneous tissues, GI, and/or mild respiratory) | Flushing, urticaria, periorbital, or facial angioedema; mild dyspnea, wheeze, or upper respiratory symptoms; mild abdominal pain and/or emesis. |
| 2. *Moderate* (mild symptoms + features suggesting moderate respiratory, cardiovascular or GI symptoms) | Marked dysphagia, hoarseness, and/or stridor; shortness of breath, wheezing, and retractions; crampy abdominal pain, recurrent vomiting, and/or diarrhea; and/or mild dizziness. |
| 3. *Severe* (hypoxia, hypotension, or neurological compromise) | Cyanosis or SpO_2_ ≤92% at any stage; hypotension^a^; confusion; collapse; loss of consciousness; or incontinence. |

^a^Systolic blood pressure: <70 mmHg in subjects aged 1 month to 1 year, <70 mmHg + [2 x age in years] in subjects aged >1 to 10 years.

GI, gastrointestinal.

# Figure Legends

## Figure E1. Summary of pooled clinical trials

^a^ARC008 includes participants from a PTAH clinical trial or a future clinical trial that identifies ARC008 as a potential post-study option in the parent protocol.

ARC003, PALISADE (NCT02635776); ARC007, RAMSES (NCT03126227); ARC010, ARTEMIS (NCT03201003); ARC004, PALISADE follow-on (NCT02993107); ARC011, RAMSES follow-on study (NCT03337542).
